# Comparative analysis of gut symbionts in *Tribolium castaneum* (Coleoptera: Tenebrionidae) and their dietary substrate, sauce-flavored Daqu

**DOI:** 10.3389/finsc.2025.1614310

**Published:** 2025-07-02

**Authors:** Jun Lü, Shan Xu, Can Teng, Rujia Huang, Guiqin Xiong, Qin Cheng

**Affiliations:** ^1^ School of Food Engineering, Moutai Institute, Renhuai, Guizhou, China; ^2^ Quality Monitoring and Evaluation Center, Moutai Institute, Renhuai, Guizhou, China

**Keywords:** sauce-flavored Daqu, *Tribolium castaneum*, gut symbionts, 16S rDNA, ITS

## Abstract

*Tribolium castaneum* (red flour beetle), a major pest infesting stored sauce-flavored Daqu (SFD), causes significant economic losses in the sauce-flavored liquor industry. This study analyzed microbial interactions between SFD and *T. castaneum* (adults and larvae) using 16S rDNA and ITS sequencing. *T. castaneum* guts primarily hosted Bacteroidota (44.7% adults, 50.9% larvae) and Proteobacteria, contrasting SFD’s Firmicutes-dominated community (89.3%), featuring *Oceanobacillus* (31.7%) and *Bacillus* (11.2%). Fungal communities across groups were Ascomycota-rich (90%), with *Aspergillus* (86%) as core, while larvae uniquely harbored *Lichtheimia* (5.5%). Larvae shared more bacterial taxa with SFD (5 genera vs. 3 in adults), yet high-abundance SFD bacteria (e.g., *Weissella*) were scarce in guts (0.6%) and vice versa. Fungal source tracking revealed SFD contributed 89–94% of gut fungi, vastly exceeding bacterial inputs (2.8–5%). Shared bacterial ASVs (n=58) exhibited functional divergence: carbohydrate metabolism dominated in SFD, whereas insect-associated ASVs enriched drug resistance genes. Findings suggest *T. castaneum* selectively colonizes SFD bacteria (e.g., *Bacillus*, *Oceanobacillus*) while proportionally acquiring fungi (e.g., *Aspergillus*) via dietary transmission. These microbes may act as a gut “seed bank” or host-selected symbionts, warranting further validation to clarify their ecological roles and inform microbially-based pest control strategies.

## 1 Introduction

Insect gut microbiota was regarded as an important extension of host physiological functions (1), playing key roles in nutritional metabolism (e.g., complex polysaccharide degradation), immune regulation (e.g., pathogen antagonism), and environmental adaptation (2, 3). Gut microbiota colonization begins during the egg stage or early hatching, and its community structure and function are co-regulated by the host’s genes, developmental stages, diet, and environmental microorganisms (4, 5). Recent studies have shown that microorganisms in the host’s food not only serve as a crucial source for gut microbiota but may also influence the host’s health and adaptability through metabolic interactions (6, 7). However, there is a lack of systematic research on the interaction mechanisms between storage pests and their food microbiota, particularly the role of fungal communities in transmission and colonization. Fungi can provide nutrients to insects and other microorganisms by breaking down complex organic matter, thus playing a crucial role in the material cycles of ecosystems (8). This decompositional function not only supports the survival of insects but may also impact the productivity and stability of the entire ecosystem.

Sauce-flavored Daqu (SFD), as the core fermentation substrate for sauce-flavored liquor, constitutes a dynamic ecosystem formed by Firmicutes, *Aspergillus*, and other complex microbial communities (9). Within SFD storage environments, SFD serves as the primary food source for *T. castaneum*, a major pest whose feeding behavior (both adults and larvae) can lead to up to 30% quality loss in SFD (10). While the ecological harm caused by this pest is widely documented, the association between its gut microbiota and food microbiota, as well as the ecological significance of this interaction, remain unclear. *T. castaneum* directly interacts with the microbial components of SFD through its consumption, providing a potential pathway for the transmission of food microorganisms to the insect’s gut. There is evidence that insects can selectively enrich low-abundance microorganisms from their environment through food consumption (e.g., bees acquire lactic acid bacteria from pollen) (11), but whether storage pests like *T. castaneum* have a similar microbial selection mechanism and how food microbiota affect gut microbiota assembly remain unclear. It is worth noting that most existing studies focus on bacterial communities, while fungi, as core functional groups in the SFD fermentation process, have long been neglected in the host-food interaction research.

Despite the known impact of *T. castaneum* on SFD quality, the factors influencing the assembly and differentiation of their gut microbiota remain poorly understood. Additionally, differences between larvae and adults in developmental stages and nutritional needs may shape the differentiation of their gut microbiota, but relevant studies are still scarce. Therefore, in this study, we used the *T. castaneum*-SFD system as a model to analyze the interaction pattern between the storage pest and its food microbiota using bacterial (16S rDNA) and fungal (ITS) amplicon sequencing. This provides a new perspective on the mechanisms of insect gut microbiota assembly and the development of green pest control strategies based on microbial regulation.

## 2 Materials and methods

### 2.1 Experimental materials

*T. castaneum* was collected from the dry Daqu warehouse of Qinghua Liquor Co., Ltd., in Moutai Town, Renhuai City. The insects were raised under conditions of 30°C ± 1°C, relative humidity RH 50% ± 10%, using SFD as the substrate.

### 2.2 Experimental methods

#### 2.2.1 Sample preparation for sequencing

After a 24-hour starvation period, one-day-old adult and last-instar larvae of *T. castaneum* were washed with 75% ethanol and then rinsed three times with distilled water. Under sterile conditions, the insect individuals were placed in 1× phosphate-buffered saline (PBS) and their entire gut was dissected with sterilized tweezers. The gut was washed three times with sterile water to remove any gut contents. Prior to DNA extraction, the insect guts were stored in 1.5 mL EP tubes containing 40 µL of sterile H_2_O at -20°C. Each group had three replicates, each consisting of 30 ~ 40 insect guts.

#### 2.2.2 Nucleic acid extraction

DNA extraction from insect guts and SFD was performed using the HiPure Tissue DNA Mini Kit (Magen, Shanghai), following the kit’s instructions. DNA concentration and purity were determined using 1% agarose gel electrophoresis and NanoDrop™ 2000 (Thermo Scientific, USA).

#### 2.2.3 Amplicon sequencing

DNA samples were sent to Shenzhen MicroKeMeng Technology Group Co., Ltd. for amplicon sequencing. The bacterial 16S rDNA V3-V4 region were amplified with the primers 341F (5’ CCTAYGGGRBGCASCAG 3’), and 806R (5’ GGACTACNNGGGTATCTAAT 3’); and fungal ITS1-ITS4 region were amplified with the primers ITS1 (5’TCCGTAGGTGAACCTTGCGG 3’) and ITS 4 (5’ TCCTCCGCTTATTGATATGC 3’). Samples were sequenced on an Illumina MiSeq platform (PE250).

#### 2.2.4 Data analysis

Raw sequencing data were processed as follows: Initially, raw data for each sample were demultiplexed based on barcodes, followed by the removal of barcodes and primers. Paired-end reads were then assembled using FLASH software (v1.2.11; http://ccb.jhu.edu/software/FLASH/) (12) to generate raw tags (Raw Tags). Quality control was performed to filter out low-quality sequences, resulting in clean tags (Clean Tags). Chimeric sequences were further removed using the UCHIME algorithm to yield high-confidence effective data (Effective Tags). Finally, the DADA2 module in QIIME2 (v2020.8; https://qiime2.org/) (13) was employed for denoising, generating Amplicon Sequence Variants (ASVs) and feature tables.

Alpha diversity indices (Chao1 and Shannon) were calculated with QIIME2 (Version 2020.8), and statistical comparisons were performed using nonparametric Kruskal-Wallis tests. The microbial community differences between groups were assessed with PLS-DA in R, using 200 permutation tests for cross-validation. Microbial source tracking was achieved with the software SourceTracker (version 1.0.1) and default parameters (sink_rarefaction_depth = 1000, smoo_env = 0.1, source_rarefaction_depth = 1000, alpha1 = 0.001). Functional diversity of bacteria and fungi was predicted using the PICRUSt2 (14) and FUNGuild (15) tools, respectively. KEGG pathway differences between samples were determined using one-way ANOVA and Tukey’s HSD test, with significance at *p* < 0.05. The analysis of the microbiome co-occurrence network was performed using the R software package “WGCNA” through CNSknowall (https://cnsknowall.com), a comprehensive web service for biomedical data analysis and visualization.

## 3 Results

### 3.1 Diversity of microbiome community

**Figure 1:**
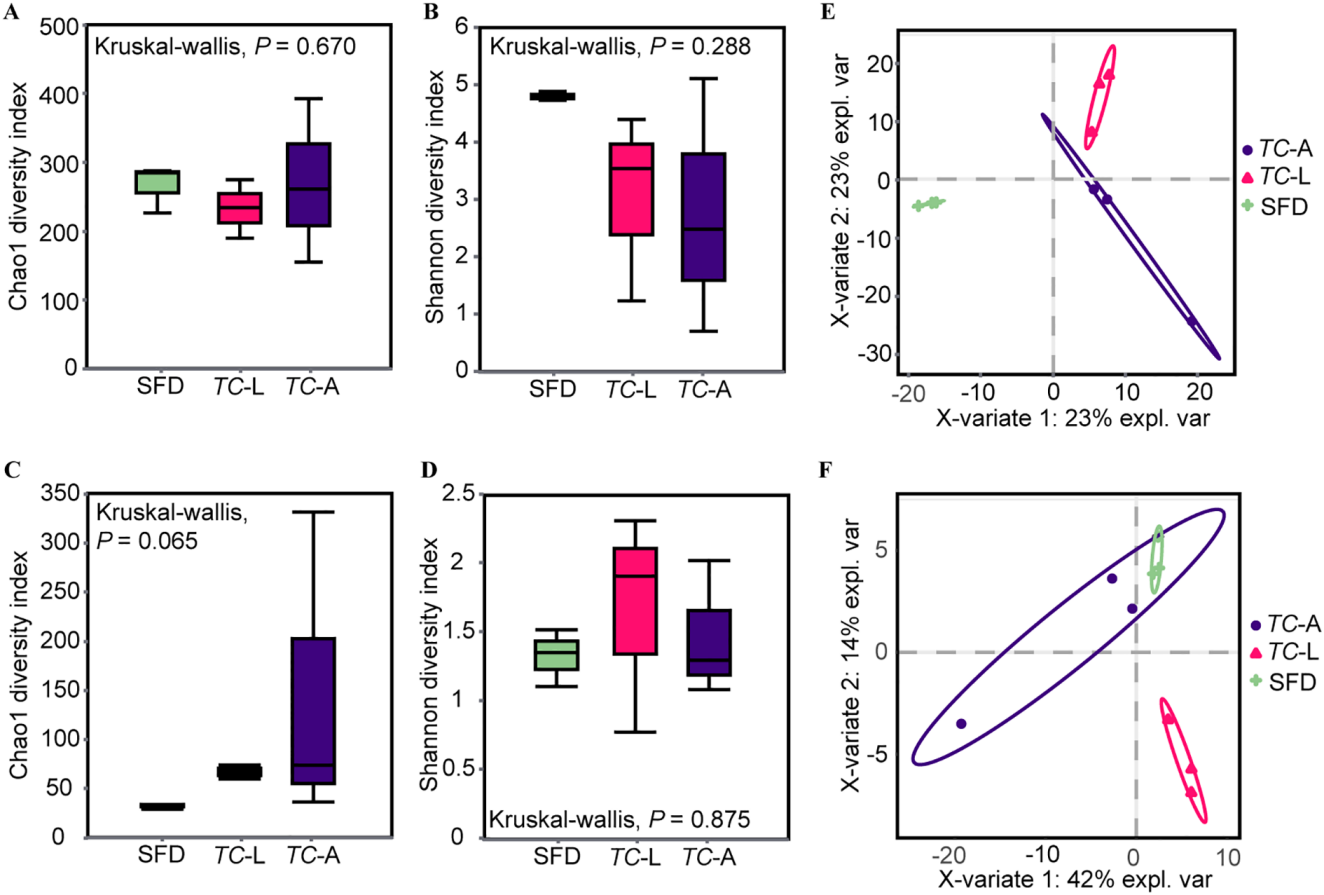
Alpha and beta diversity of microbiota. Alpha diversity is based on the Shannon diversity index and Chao1 index. **(A)** Bacterial Chao1 diversity; **(B)** Bacterial Shannon diversity; **(C)** Fungal Chao1 diversity; **(D)** Fungal Shannon diversity. Partial least squares discriminant analysis (PLSDA) of the microbiota in each group. **(E)** Bacteria; **(F)** Fungi. Kruskal-Wallis test was used in the statistical test of the alpha diversity (no statistically significant difference found). SFD, Sauce-flavored Daqu; *TC*-L, *Tribolium castaneum* larva; *TC*-A, *T. castaneum* adult.

Amplicon sequencing produced 793,606 high-quality 16S rDNA sequences with 1,242 ASVs, and 847,699 high-quality ITS sequences with 588 ASVs. Alpha diversity indices, including Chao1 and Shannon indices, were estimated for the bacterial and fungal communities in *T. castaneum* and SFD. The results showed no significant differences in bacterial and fungal community diversity between *T. castaneum* adults, larvae, and SFD (**Figures 1A–D**). Using Partial Least Squares Discriminant Analysis (PLS-DA), we analyzed the differences in microbial communities among the three groups. PLS-DA showed that microbial community structures differed significantly between the treatment groups. For bacteria, SFD was clearly separated from both adult and larval *T. castaneum* along PC1, while adults and larvae were notably separated along PC2 (**Figure 1E**). For fungal communities, we observed an overlap between adult *T. castaneum* and SFD, while larvae were distinctly separated from both (**Figure 1F**).

### 3.2 Microbial composition

**Figure 2:**
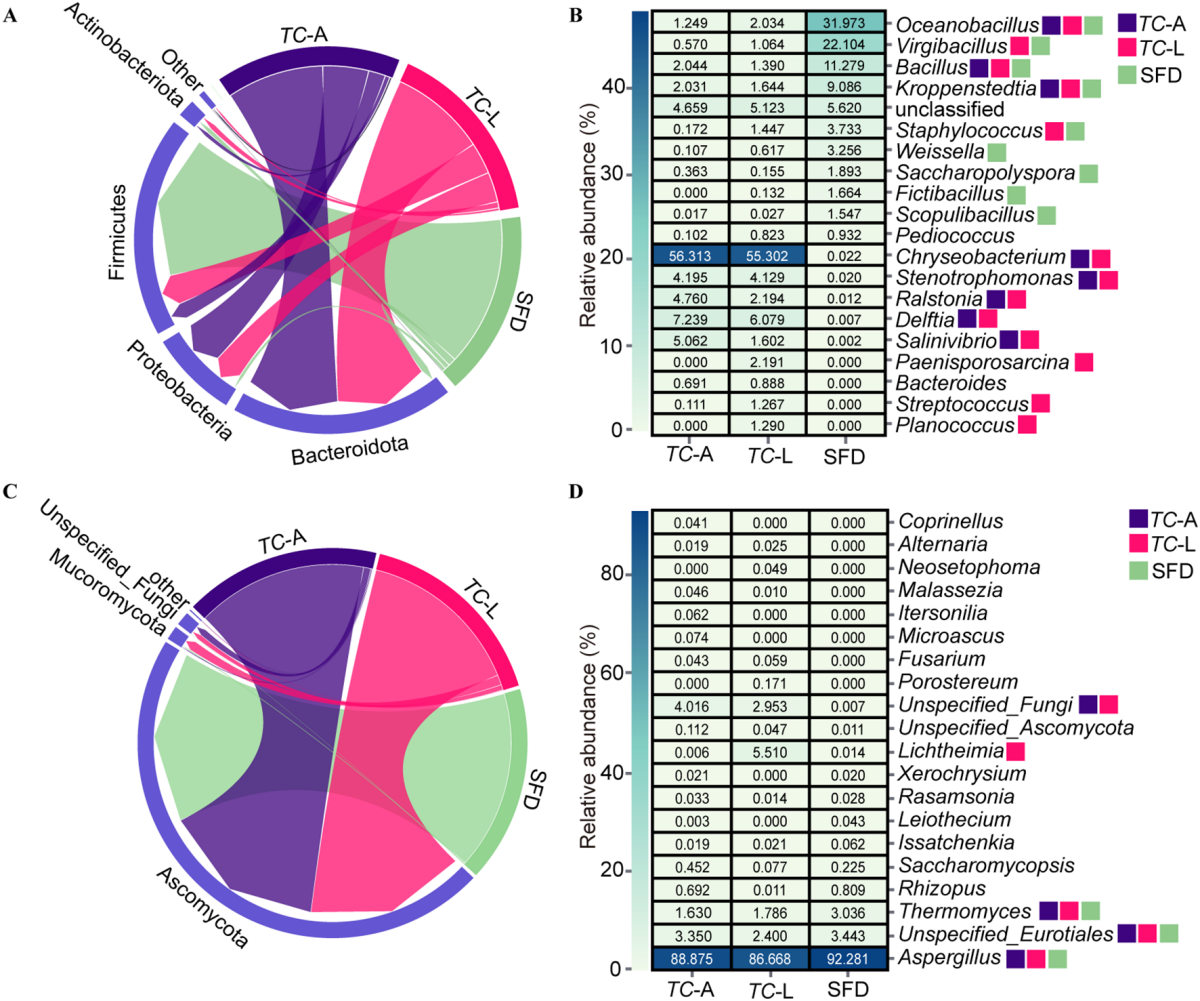
Composition analysis of microorganisms. **(A)** Relative abundances of main bacterial phyla. **(B)** Heatmap showing the top 20 abundant bacterial genera. **(C)** Relative abundances of main fungal phyla. **(D)** Heatmap showing the top 20 abundant fungal genera. Colored squares indicate core microbes in different groups.

The dominant bacterial phyla in the adult *T. castaneum* gut were Bacteroidota (58.1%), Proteobacteria (27.5%), Firmicutes (9.2%), and Actinobacteriota (2.8%) (**Figure 2A**). In the larval gut, the dominant bacterial phyla were Bacteroidota (57.6%), Firmicutes (19.0%), Proteobacteria (18.0%), and Actinobacteriota (3.7%) (**Figure 2A**). At the genus level, the dominant bacterial genera in adult *T. castaneum* were *Chryseobacterium* (56.3%), *Delftia* (7.2%), *Salinivibrio* (5.1%) (**Figure 2B**). The dominant bacterial genera in the larval gut were *Chryseobacterium* (55.3%), *Delftia* (6.1%), *Stenotrophomonas* (4.1%), and *Ralstonia* (2.2%) (**Figure 2B**). In contrast, the microbial composition of SFD differed significantly from that of *T. castaneum*. The bacterial community in SFD was dominated by Firmicutes (89.9%), with genera such as *Oceanobacillus* (32.0%), *Virgibacillus* (22.1%), and *Bacillus* (11.3%) (**Figures 2A, B**).

The dominant fungal phylum in both adult and larval was Ascomycota (95.0% and 91.3%, respectively) (**Figure 2C**). At the genus level, the dominant fungal genera in the adult gut were *Aspergillus* (88.9%) and *Thermomyces* (1.6%), while in larval, the genera were *Aspergillus* (86.7%), *Lichtheimia* (5.5%), and *Thermomyces* (1.8%) (**Figure 2D**). The fungal composition in SFD was similar to that in the adult gut, mainly consisting of *Aspergillus* (92.3%) and *Thermomyces* (3.0%) (**Figure 2D**).

Based on the criterion of average relative abundance >1%, we defined the distribution pattern of core genera. For bacteria (**Figure 2B**), 9 core genera were identified in SFD, while 8 and 13 core genera were found in the adult and larval guts, respectively. The adult gut shared 3 core genera with SFD (*Oceanobacillus*, *Bacillus*, and *Kroppenstedtia*), while the larval gut shared 5 core genera with SFD (*Oceanobacillus*, *Virgibacillus*, *Bacillus*, *Kroppenstedtia*, and *Staphylococcus*), indicating that larvae have a stronger ability to colonize bacterial species from SFD. However, there were also significant differences between the communities: 4 core genera (*Weissella*, *Saccharopolyspora*, *Fictibacillus*, and *Scopulibacillus*) present in SFD were found at very low abundance (0.6%) in *T. castaneum* guts, while 5 core genera (*Chryseobacterium*, *Delftia*, *Stenotrophomonas*, *Ralstonia*, and *Salinivibrio*) were almost undetectable in SFD (0.05% average abundance). In contrast to bacteria, the core fungal genera across the three groups were more uniform, with *Aspergillus* and *Thermomyces* being core genera in both SFD and the adult gut, while the larval gut had an additional core genus, *Lichtheimia* (**Figure 2D**).

### 3.3 Analysis of shared microbiota

**Figure 3:**
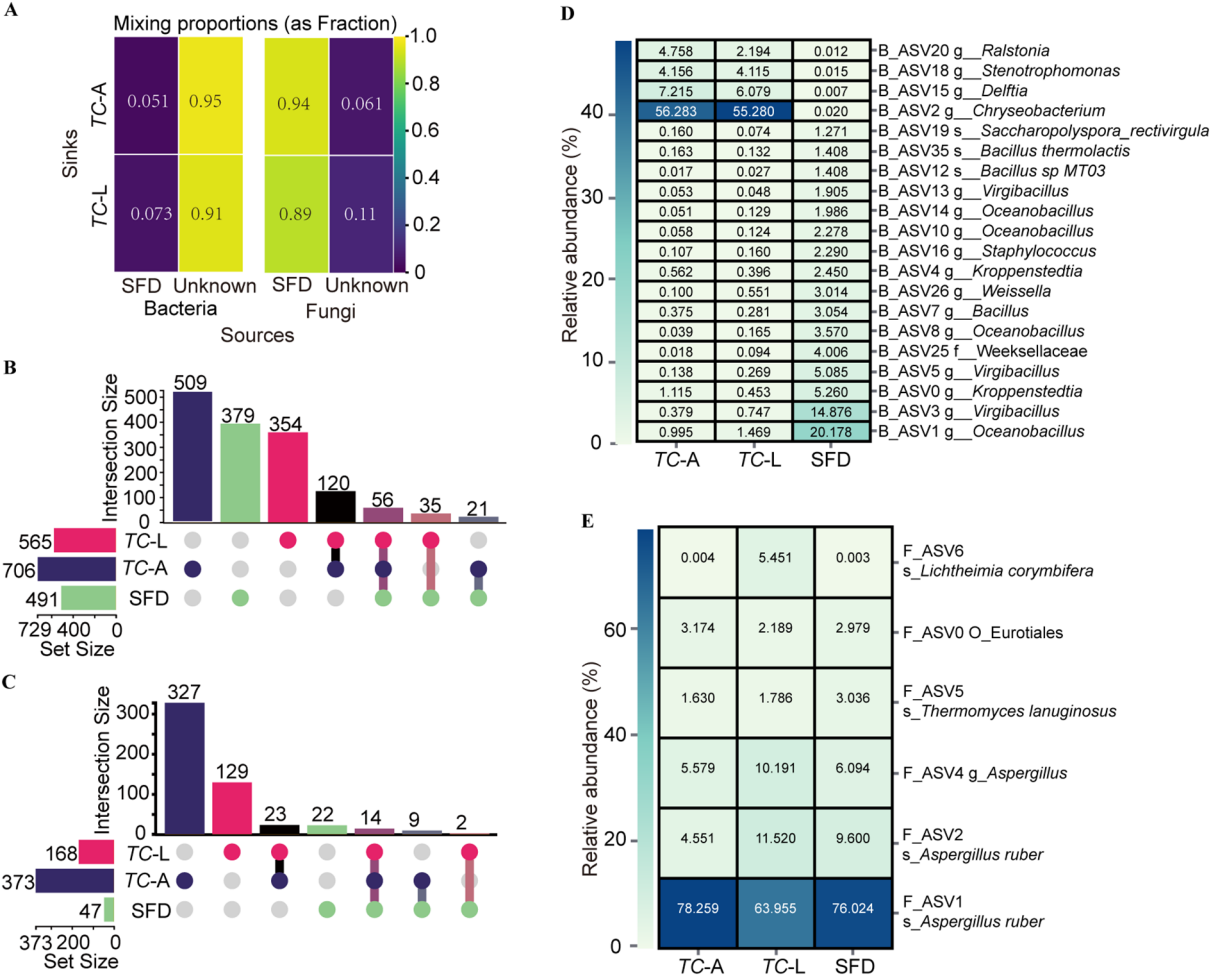
Shared microbial analysis between SFD and *T. castaneum*. **(A)** Contribution of SFD to the gut microorganisms of *T. castaneum.* **(B)** Bacterial and **(C)** Fungal upset plot. **(D)** Heatmap of the core ASV screened from the 58 shared bacterial ASV (B_ASV). **(E)** Heatmap of the core ASV screened from the 14 shared fungal ASV (F_ASV).

Food is a major route through which insects acquire microbiota (16). To analyze the contribution of SFD to the gut microbiota of adult and larval *T. castaneum*, we performed microbial source tracking analysis using SourceTracker. The results showed that the contribution of SFD to the bacterial microbiota in adult and larval *T. castaneum* was 5.1% and 7.3%, respectively (**Figure 3A**). For fungi, SFD contributed 94% and 89% to the fungal microbiota in adult and larval *T. castaneum*, respectively (**Figure 3A**).

For bacteria (**Figure 3B**), we identified 1,242 unique bacterial ASVs. Specifically, we found 565 ASVs in larvae, 706 in adults, and 491 in SFD, with 35 ASVs present only in SFD and larvae, 21 ASVs present only in SFD and adults, and 56 ASVs shared across all three groups. For fungi (**Figure 3C**), we identified 588 unique fungal ASVs. Specifically, we found 168 ASVs in larvae, 373 in adults, and 47 in SFD, with 2 ASVs found only in SFD and larvae, 9 ASVs found only in SFD and adults, and 14 ASVs shared across all three groups.

Among the 56 shared bacterial ASVs (B_ASVs), we evaluated core ASVs (relative abundance >1%) across groups (**Figure 3D**). The analysis identified 5 core B_ASVs in adults, with B_ASV0 overlapping with SFD, and 5 core B_ASVs in larvae, with B_ASV1 overlapping with SFD. Notably, SFD harbored 16 core bacterial B_ASVs. For fungi (**Figure 3E**), the 14 shared fungi ASVs (F_ASVs) included 5 core F_ASVs in adults, 6 in larvae, and 5 in SFD. Strikingly, F_ASV1 emerged as the most abundant F_ASV across all three groups, underscoring its potential ecological significance in both *T. castaneum* and SFD microbial networks.

### 3.4 Analysis of shared microbiota roles and interaction networks

**Figure 4:**
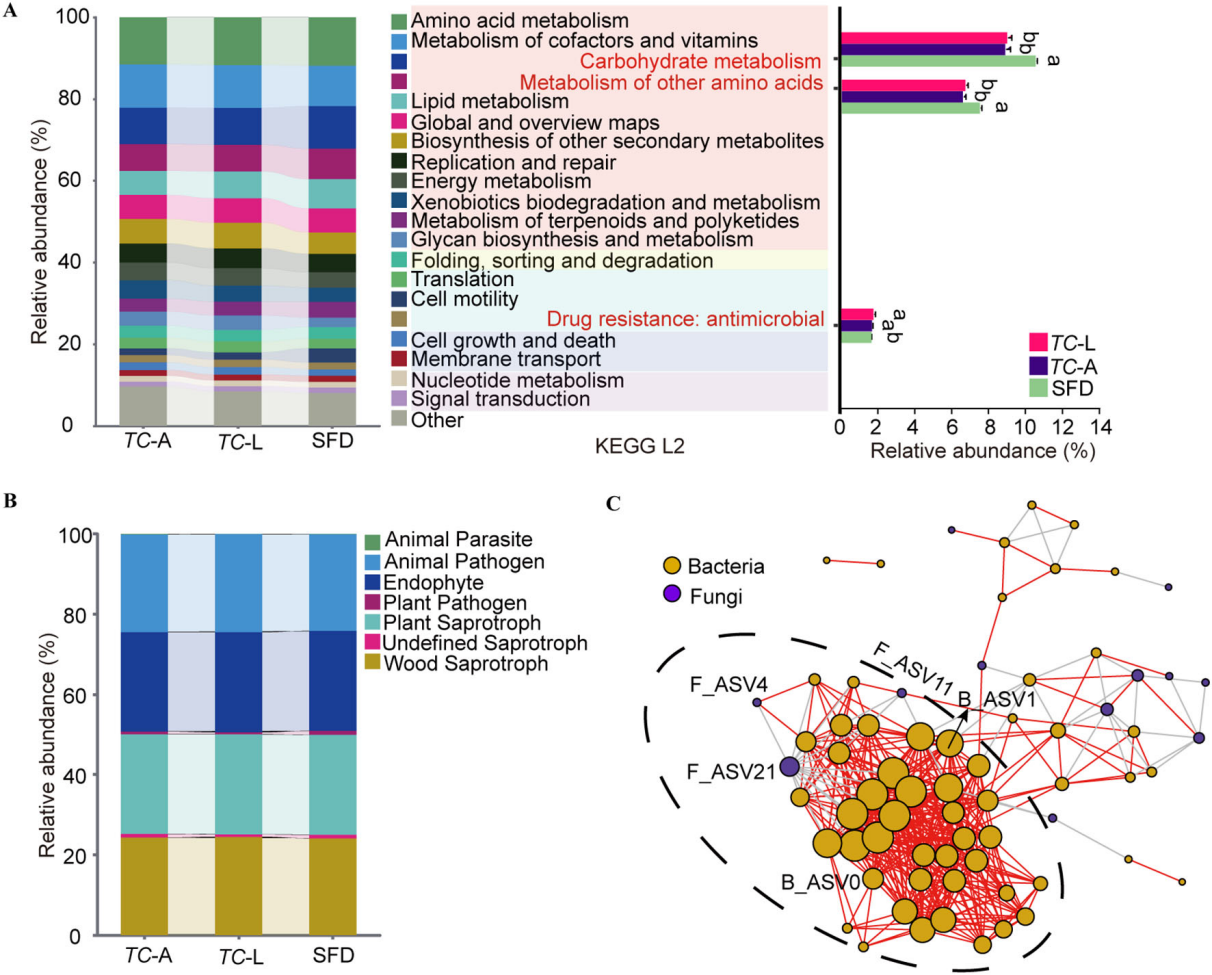
Functional and collinearity analysis of shared microorganisms. **(A)** PICRUSt2 analyses predicting ecological functions of shared bacteria. Different lowercase letters represent significant differences (*p* < 0.05, Tukey’s HSD). **(B)** FUNGuild analyses predicting ecological functions of shared fungi. **(C)** Microbiota network patterns of shared microorganisms in *T. castaneum*. Network has been drawn based on Spearman’s rank correlation. The significance was set at *p* < 0.05 and the threshold of correlation was set asR= 0.65. In the network, the vertexes (circles) correspond to microbial species while the weight of the edges (segments) represents correlations between microbes. Positive correlations are colored in red while negative correlations are colored in gray. Orange circles represent bacteria, and purple circles represent fungi.

*T. castaneum* shared 56 bacterial ASVs with SFD, which were classified into 3 phyla and 26 genera, with *Bacillus* being the most abundant, followed by *Oceanobacillus* and *Kroppenstedtia* (**Table 1**). Using functional predictions for these 58 ASVs, we found that they were enriched in six first-level pathways (Cellular Processes, Environmental Information Processing, Genetic Information Processing, Human Diseases, Metabolism, Organismal Systems) and 44 second-level pathways. Among these, pathways related to carbohydrate metabolism and metabolism of other amino acids were enriched in SFD, while pathways related to drug resistance: antineoplastic were significantly enriched in *T. castaneum* (**Figure 4A**).

**Table 1:** Shared ASVs between the SFD and *T. castaneum.*.

Kingdom	Phylum	Genus	ASV
Bacteria	unidentified	unidentified	ASV228
	Actinobacteriota	unidentified_Pseudonocardiaceae	ASV32
		*Saccharopolyspora*	ASV19 ASV34
		*Streptomyces*	ASV98
	Bacteroidota	unidentified_Weeksellaceae	ASV25
		*Chryseobacterium*	ASV2
	Firmicutes	*Bacillus*	ASV7 ASV23 ASV44 ASV45 ASV59 ASV74 ASV31 ASV38 ASV35 ASV48 ASV78
		*Oceanobacillus*	ASV1 ASV8 ASV10 ASV14 ASV21
		*Virgibacillus*	ASV3 ASV5 ASV13
		*Scopulibacillus*	ASV12
		*Enterococcus*	ASV216
		*Companilactobacillus*	ASV106 ASV329
		*Lactobacillus*	ASV57
		*Latilactobacillus*	ASV84 ASV339
		*Ligilactobacillus*	ASV107
		*Limosilactobacillus*	ASV83
		*Pediococcus*	ASV33 ASV69
		*Weissella*	ASV26
		*Staphylococcus*	ASV16 ASV52 ASV49
		*Kroppenstedtia*	ASV88 ASV0 ASV4 ASV36 ASV43
		*Novibacillus*	ASV289
		*Thermoactinomyces*	ASV54 ASV138
		*Megasphaera*	ASV636
	Proteobacteria	*Acetobacter*	ASV61
		unidentified_Mitochondria	ASV465
		*Ralstonia*	ASV20
		*Delftia*	ASV15
		*Escherichia-Shigella*	ASV123
		*Stenotrophomonas*	ASV18
Fungi	Ascomycota	*Aspergillus*	ASV1 ASV2 ASV4 ASV15 ASV17 ASV21 ASV34
		*Rasamsonia*	ASV35
		*Saccharomycopsis*	ASV11
		*Thermomyces*	ASV5
		unidentified_Eurotiales	ASV0 ASV3
	Mucoromycota	*Lichtheimia*	ASV6
		*Rhizopus*	ASV9

*T. castaneum* shared 14 fungal ASVs with SFD, which were classified into 2 phyla and 7 genera of fungi (**Table 1**). To elucidate their primary functions, the potential roles of each ASV were characterized utilizing FUNGuild. Among the 14 ASVs analyzed, 11 exhibited highly probable or probable life strategies, with predominant functions identified as animal pathogens, endophytes, plant saprotrophs, and wood saprotrophs (**Figure 4B**).

To understand the interactions among the shared microorganisms in the *T. castaneum* gut, we established a microbial symbiotic network (**Figure 4C**), which included 70 nodes and 457 edges, of which 407 were positive correlations and 50 were negative correlations. Network analysis revealed a highly dense subcluster containing 42 bacterial nodes and 3 fungal nodes, suggesting strong symbiotic relationships between these microorganisms. Notably, only B_ASV0 and B_ASV1 from the core bacterial ASVs were located in this subcluster, while only F_ASV4 from the core fungal ASVs was involved, indicating that these core microbes may play a key role in maintaining community stability. Additionally, fungal F_ASV21 was significantly negatively correlated with 15 bacterial nodes, suggesting an antagonistic relationship, possibly influencing bacterial colonization and growth through resource competition or the production of inhibitory metabolites.

## 4 Discussion

The gut microbiota of adult and larval *T. castaneum* is dominated by Firmicutes, Proteobacteria, and Bacteroidota (**Figure 2A**), resembling the gut microbiota of many herbivorous insects and mammals (17). Firmicutes plays a crucial role in degrading complex carbohydrates, while Bacteroidota breaks down plant polysaccharides, enhancing digestion (18). Proteobacteria aids nitrogen fixation and metabolism, supporting host health (5). These microbes enable *T. castaneum* to efficiently digest SFD. Notably, *Chryseobacterium* is the dominant genus in both life stages (relative abundance >40%, **Figure 2B**), and its cellulolytic properties (19), common in xylophagous insects (20, 21) suggest its role in adapting to the high-carbohydrate SFD diet. *Oceanobacillus* is the predominant bacterial genus in SFD (**Figure 2B**), significantly impacting its acidity and esterification capacity through the accumulation of metabolites, which in turn influences the final quality of Baijiu (22). The fungal community in SFD shares *Aspergillus* (Ascomycota) as a core genus (**Figures 2C, D**), aiding cellulose degradation in termite guts (23). Larvae share more core bacterial genera with SFD than adults (5 vs. 3), indicating stronger microbial selection in larvae, likely due to higher nutritional demands.

Insects predominantly acquire their microbiota from dietary sources (16), as demonstrated by bees obtaining microbiota from floral environments (24, 25). However, our study indicates a minimal bacterial contribution from SFD to the guts of *T. castaneum*, with only 5.1% in adults and 7.3% in larvae, in stark contrast to fungal contributions exceeding 89% (**Figure 3A**). This observation is consistent with findings in other insects, where gut bacteria are not primarily derived from the diet (26, 27). The observed discrepancy likely results from *T. castaneum*’s selective colonization of environmental microbes, as evidenced by the predominance of *Chryseobacterium* in the guts (40–50%) despite its low abundance in SFD (0.022%). Such selective enrichment may reflect host nutritional requirements or gut-specific conditions that favor bacterial proliferation. Similar mechanisms are observed in mammals; for instance, Tibetan macaques (*Macaca thibetana*) selectively retain rare soil bacteria in their guts despite their low environmental abundance (28). Additionally, the pika (*Ochotona* spp.) gut primarily selects for low-abundance but diverse environmental bacteria in a host species-specific manner (29).

The shared microbiota between SFD and *T. castaneum* presents a promising opportunity for RNA interference (RNAi)-based pest control. Gene editing could engineer these microbes to express double-stranded RNA (dsRNA) targeting lethal genes in *T. castaneum*. When consumed, the dsRNA would silence essential genes, providing effective pest control. ASV0 and ASV1 in bacterial microbiota, core to both SFD and *T. castaneum*, may be prime candidates for this strategy (**Figure 3D**). The shared microbiota also forms an extensive interaction network (**Figure 4C**), enabling microbiome-based interventions that competitively exclude harmful gut bacteria in pests, offering a green pest control solution.

Though *T. castaneum* is typically a pest in SFD, causing quality loss and altering physicochemical properties (e.g., moisture, starch, acidity, saccharification, liquefaction, fermentation) (10), the discovery that over 89% of its fungi are shared with SFD provides new insights into its role. *T. castaneum* may function as a fungal conveyor, dispersing fungi within SFD and enhancing fungal uniformity.

In summary, the assembly of *T. castaneum’*s gut microbiota is influenced by both host selection and interactions with food-associated microbes. The efficient transfer of fungi offers a novel perspective on insect-food microbiota interactions, paving the way for innovative pest control and microbial resource utilization in the sauce-flavored liquor industry.

## 5 Conclusions

The findings indicate that *T. castaneum* selectively colonizes specific microbes, particularly fungi, from SFD. The high fungal transmission efficiency offers novel insights into pest-microbe interactions and suggests leveraging shared microbiota for RNAi-based pest control strategies. Future studies should validate whether these microbes represent transient passengers or stable functional units, and explore their roles in enhancing SFD fermentation or pest resilience. This work provides a theoretical foundation for green pest management and microbial resource utilization in the sauce-flavored liquor industry.

## Data Availability

The raw data supporting the conclusions of this article will be made available by the authors, without undue reservation.
